# Predictive values of procalcitonin for coinfections in patients with COVID-19: a systematic review and meta-analysis

**DOI:** 10.1186/s12985-023-02042-x

**Published:** 2023-05-08

**Authors:** Shanchen Wei, Lina Wang, Lianjun Lin, Xinmin Liu

**Affiliations:** Department of Geriatrics, Peking University First Hospital, Xishiku Avenue No 8, Xicheng District, Beijing, 100034 China

**Keywords:** Procalcitonin, Coinfections, Predictive, COVID-19, Meta-analysis

## Abstract

**Objectives:**

To assess the ability of procalcitonin (PCT)—a promising marker for coinfections—to predict coinfections in patients with COVID-19.

**Methods:**

In this systematic review and meta-analysis, PubMed, Embase, Web of Science, Cochrane, the China National Knowledge Infrastructure (CNKI), and Wanfang were searched to identify eligible studies (up to August 30, 2021). Articles that reported the predictive value of PCT for coinfections in patients with COVID-19 were included. Individual and pooled sensitivities and specificities were reported, and *I*^2^ was used to test heterogeneity. This study was prospectively registered on the International Prospective Register of Systematic Reviews (PROSPERO) database (registration number: CRD42021283344).

**Results:**

Five studies involving a total of 2775 patients reported the predictive value of PCT for coinfections in patients with COVID-19. The sensitivity, specificity, and area under the curve of PCT in predicting coinfections in the pooled studies were 0.60 (95% CI 0.35–0.81, *I*^2^ = 88.85), 0.71 (95% CI 0.58–0.81, *I*^2^ = 87.82), and 0.72(95% CI 0.68–0.76) respectively.

**Conclusions:**

Although PCT has limited predictive value for coinfections in patients with COVID-19, lower PCT levels seem to indicate a decreased probability of having a coinfection.

**Supplementary Information:**

The online version contains supplementary material available at 10.1186/s12985-023-02042-x.

## Introduction

The prevalence of bacterial coinfections in hospitalized patients with coronavirus disease 2019 (COVID-19), a novel human-to-human infectious disease [1], is less than 10% [2–4]. The rate of bacterial respiratory infection in critically ill patients with COVID-19 has been estimated to be between 14 and 28% [5–7], and the prevalence of coinfections in patients in intensive care units (ICUs) ranges from 14 to 50% [3, 8]. However, another study found that 81.7% of patients who died from COVID-19 had bacterial coinfections [9]. Additionally, Martins-Filho et al. showed that sepsis was associated with a 2.4-fold increased risk of death in these patients [10]. These findings indicate that the overall rate of confirmed coinfections is low, but the mortality rate of coinfections is high.

The vast majority (57–86%) of patients with COVID-19 receive empiric antibiotic therapy [2, 4, 11], which may not be required in most cases. Antimicrobial prescriptions have increased since the pandemic began, posing the threat of increasing antimicrobial resistance worldwide [12]. Therefore, identifying an indicator that can predict COVID-19 coinfections is of important clinical significance.

Serum procalcitonin (PCT) may help identify coinfections in patients with COVID-19 [13], facilitating decisions about antibiotic therapy for lower respiratory tract infections [14–16]. However, previous studies demonstrated that in isolated COVID-19 patients, as in other viral infections, PCT levels generally remain normal (≤ 0.5 µg/L); this may be because the virus stimulates macrophages to produce interferon-γ, thereby suppressing TNF-α during the immune response [16]. Other research found that a PCT level of < 0.25 µg/L had a negative predictive value of 81%, and a PCT level of > 1 µg/L had a positive predictive value of 93% for coinfections [8].

To more rigorously assess the predictive value of PCT for coinfections in patients with COVID-19, we performed a systematic review and meta-analysis.

## Materials and methods

This meta-analysis was performed following the Preferred Reporting Items for Systematic Reviews and Meta-Analyses (PRISMA statement) guidelines [17] and was prospectively registered on the International Prospective Register of Systematic Reviews (PROSPERO) database (registration number: CRD42021283344).

### Search strategy and selection criteria

The PubMed, Embase, Web of Science, Cochrane, China National Knowledge Infrastructure (CNKI), and Wanfang databases (up to August 30, 2021) were searched with the following terms: ((((((((((co-infection) OR (coinfection)) OR (super-infection)) OR (superinfection)) OR (secondary infection)) OR (bacterial infection)) OR (bacterial culture)) OR (other pathogens NOT SARS Cov-2)) OR (other organisms NOT SARS Cov-2)) AND ((procalcitonin) OR (PCT))) AND (((((Coronavirus disease 2019) OR (2019 Novel Coronavirus)) OR (SARS-CoV-2)) OR (2019-nCoV)) OR (COVID-19)).

The full search strategies are shown in Additional file 1. No language restrictions were applied. To identify additional literature, the reference lists of eligible studies and previous evidence summaries were also reviewed by two reviewers (SCW and LNW) independently. Disagreements were resolved by consensus, and in cases of persistent disagreement, the third reviewer (XML) was consulted.

The inclusion criteria of studies were as follows: (1) the predictive value of PCT for coinfections in patients with COVID-19 was evaluated; (2) a 2 × 2 table of results was able to be constructed (i.e., sufficient information was included to calculate the true positive [TP], false positive [FP], false negative [FN], and true negative [TN]). The following study types were excluded: case reports, reviews, editorials, conference abstracts, comments, letters, and animal studies.

### Data extraction and quality assessment

Relevant information was extracted from individual studies with a standardized form; specifically, the first author, publication year, number of patients (male/female), mean age, cut-off value, area under the curve (AUC), TP, TN, FP, FN, sensitivity (SEN), and specificity (SPE) were recorded. Data extraction was assessed by two reviewers (SCW and LNW), and disagreements were resolved by consensus. The Quality Assessment of Diagnostic Accuracy Studies 2 (QUADAS-2) criteria was used to evaluate each of the included studies (Additional file 2).

### Statistical analysis

StataMP (version 16.0) with the MIDAS module was used to conduct the statistical analyses. The pooled SEN, SPE, likelihood ratio (LR), and diagnostic odds ratio (DOR) with corresponding 95% confidence intervals (CIs) were calculated by a bivariate random effects meta-analysis model [18]. The extent of heterogeneity among the studies was quantified by calculating the *I*^*2*^* statistic*, and *I*^*2*^ values above 50% indicate substantial heterogeneity. The overall diagnostic accuracy was assessed by a summary receiver operating characteristic (SROC) curve. A Fagan nomograph was used to explore the relationship between the pretest probability, likelihood ratio, and post-test probability.

## Results

### Selection and characteristics of studies

**Fig. 1:**
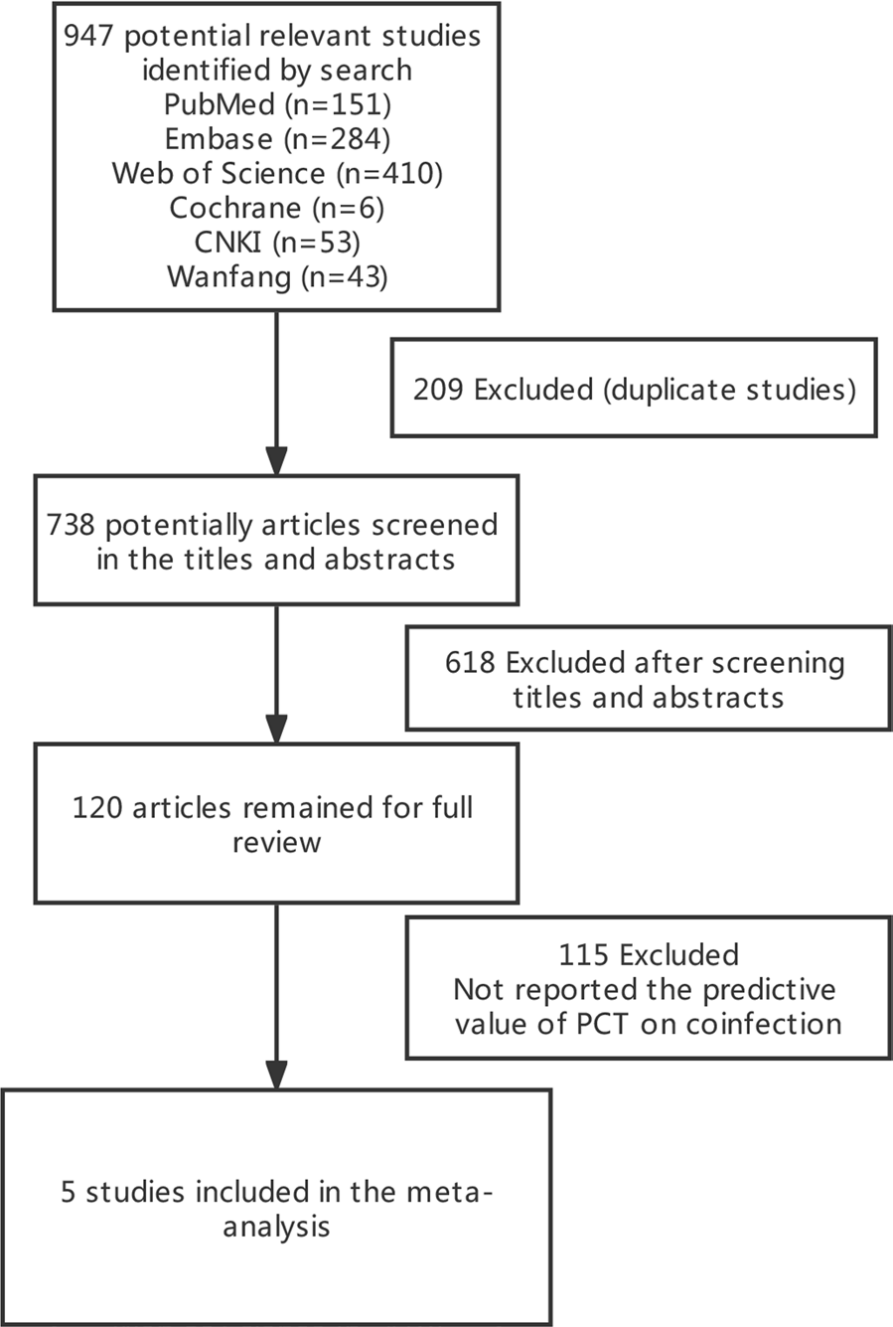
Flow diagram for the identification of eligible studies

The literature search identified 947 studies, including 151 from PubMed, 284 from Embase, 410 from Web of Science, 6 from Cochrane, 53 from the CNKI, and 43 from the Wanfang database. Figure 1 shows the study selection process. A total of 209 duplicate publications were excluded, and 618 studies were excluded after the title and abstract assessment according to the inclusion and exclusion criteria. The remaining 120 studies were reviewed by reading the full text. Of these, five studies were finally included in the meta-analysis.

The characteristics of the included studies and the predictive value of PCT for coinfections in each study are listed in Table 1. The number of participants ranged from 66 to 2443. Notably, the SEN, SPE, and AUC varied widely among the included studies. All studies were retrospective except for one [19]. All but two studies [19, 20] diagnosed coinfections with positive blood and/or lower respiratory tract cultures. One study did not specify which specimens were cultured and defined colonization as a positive culture without clinical manifestations [19]. The other study included urine cultures in addition to blood and lower respiratory tract specimens [20].

**Table 1:** Characteristics of the included studies and diagnostic test performance of procalcitonin (PCT) for coinfections

Study	Country	No. of patients	Male/female	Median age (IQR)	Cut-off (μg/L)	AUC	TP	FP	FN	TN	SEN (%)	SPE (%)	PPV (%)	NPV (%)
Alberto Dolci 2021	Italy	83	68/15	64 (53.3–72.0)	0.80	0.67	20	14	13	36	0.61	0.72	0.59	0.73
Charlotte Vanhomwegen 2021	Belgium	66	41/25	61 (49–71)	0.50	0.68	5	34	2	25	0.71	0.42	0.13	0.93
Emma J. Kooistra 2021	The Netherlands	84	58/26	NA	0.50	NA	10	8	28	38	0.26	0.83	0.56	0.58
Isabell Pink 2021	Germany	99	72/27	57 (18–91)	0.55	0.88	29	13	3	54	0.91	0.81	0.69	0.95
Michael May 2021	USA	2443	1395/1048	NA	0.50	NA	63	653	85	1642	0.43	0.72	0.09	0.95

AUC, area under the curve; *TP*, true positive; FP, false positive; FN, false negative; TN, true negative; SEN, sensitivity; SPE, specificity; NA, not available

### Predictive value of PCT for coinfections

**Fig. 2:**
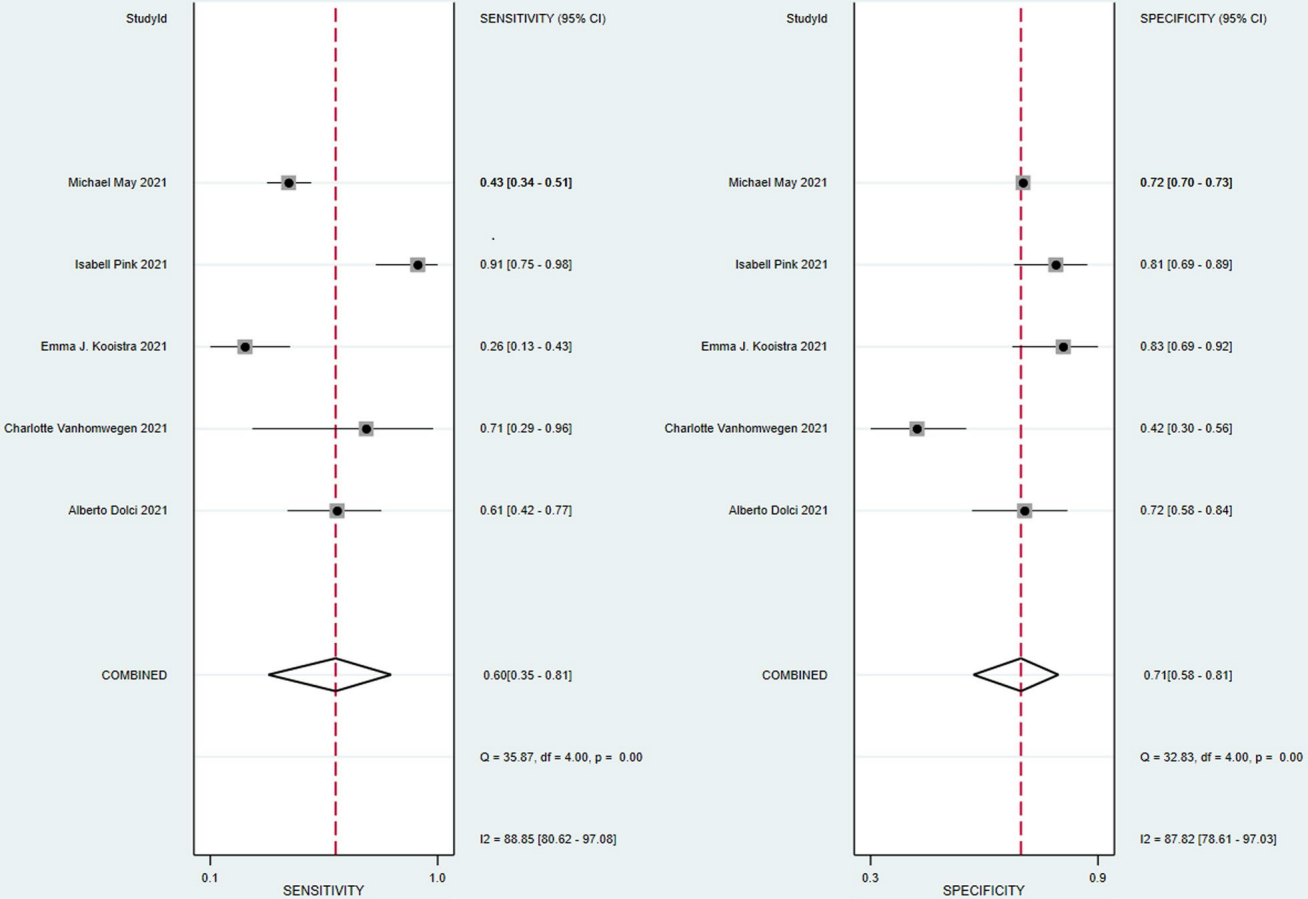
Forest plot of the sensitivity and specificity of PCT for predicting coinfection in patients with COVID-19. The pooled sensitivity and specificity were 0.60 (95% CI 0.35–0.81) and 0.71 (95% CI 0.58–0.81), respectively

**Fig. 3:**
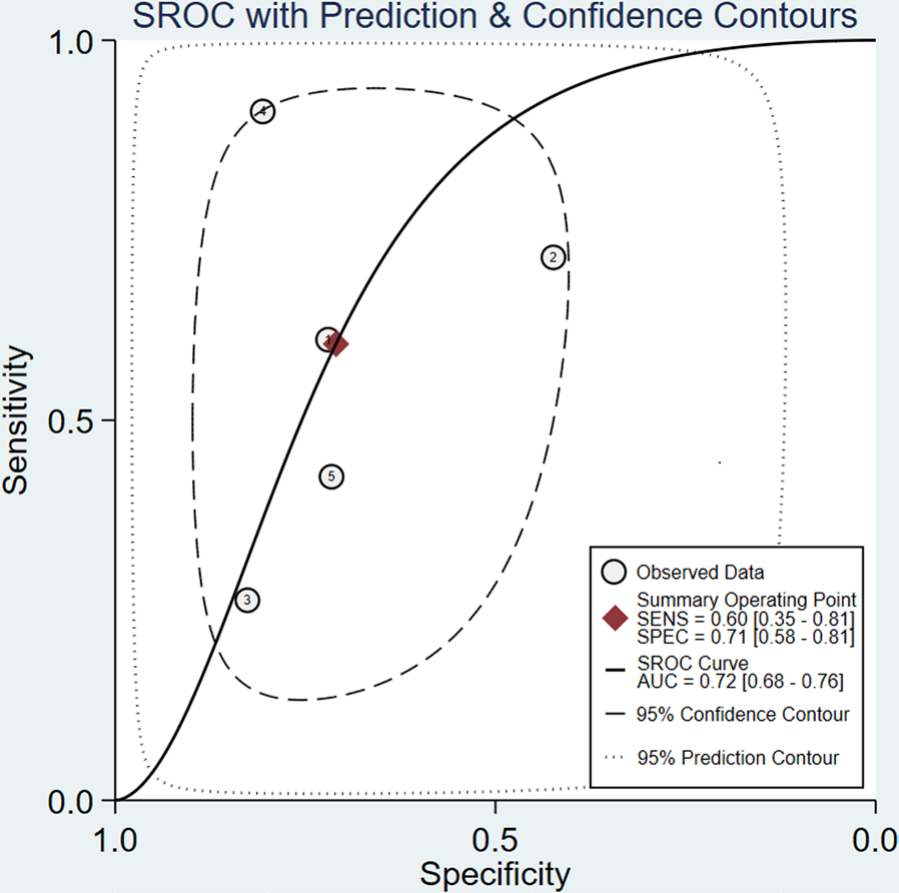
Summary receiver operating characteristic graph for the included studies. The AUC of PCT for predicting coinfection was 0.72 (95% CI 0.68–0.76)

**Fig. 4:**
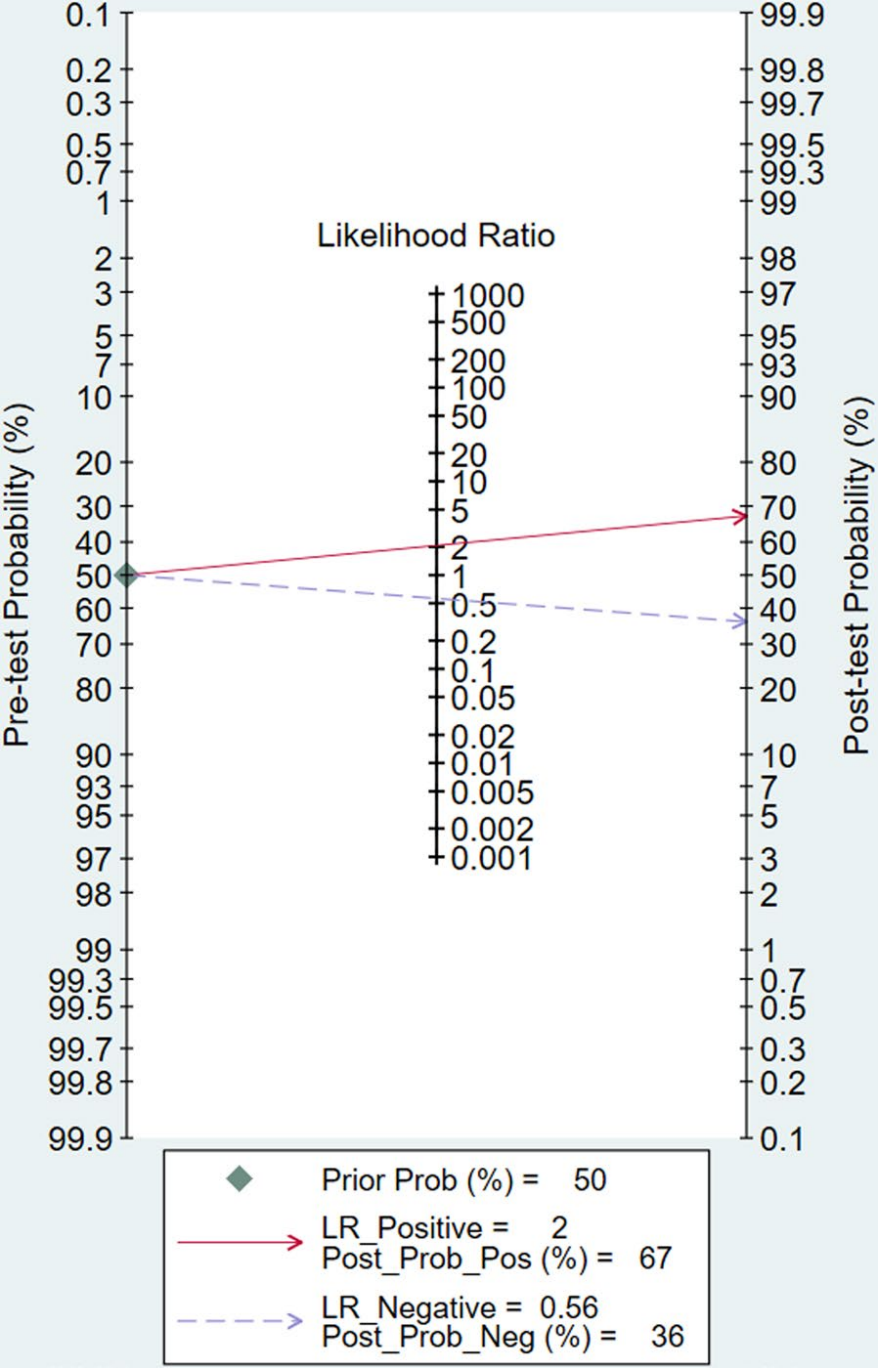
Fagan nomogram of PCT for predicting coinfection in patients with COVID-19. The pre-test probability was set to 50%. The post-test probability of PCT for the detection of coinfection was 67% when the PCT was above the cut-off value. The post-test probability was 36% when the PCT was below the cut-off value

Five studies involving a total of 2775 participants reported the predictive value of PCT for coinfections in patients with COVID-19. The combined SEN and SPE were 0.60 (95% CI 0.35–0.81, *I*^*2*^ = 88.85) and 0.71 (95% CI 0.58–0.81, *I*^*2*^ = 87.82), respectively (Fig. 2). The positive likelihood ratio was 2.1 (95% CI 1.2–3.5), and the negative likelihood ratio was 0.56 (95% CI 0.31–1.04). The DOR was 13 (95% CI 9–18). The SROC curve is shown in Fig. 3; the AUC of PCT for predicting coinfections with COVID-19 was 0.72 (95% CI 0.68–0.76), indicating limited diagnostic value of PCT. The Fagan nomogram (Fig. 4) indicated that if the pretest probability was set to 50%, the post-test probability of PCT for predicting coinfections was 67% when PCT was above the cut-off value. Conversely, when PCT was below the cut-off value, the post-test probability was 36%.

### Study quality

The methodological quality of the included studies is summarized in Additional file 2. One study only selected patients admitted to the ICU [21], and another included only critically ill patients with COVID-19 [19]. Therefore, these two studies were considered to have a high risk of patient selection bias.

## Discussion

The concentration of procalcitonin (PCT) in the circulation is low (≤ 0.1 µg/L), and an increase in PCT concentration is positively associated with the severity of bacterial infections; notably, PCT is the most sensitive indicator for the early clinical identification of bacterial and viral infections [22]. The presence of PCT had a high negative predictive value (94%) for bacterial coinfections in patients with influenza in ICUs [23]. However, the PCT concentration does not increase (≤ 0 5 µg/L) in most patients with COVID-19, but it increases frequently in severe cases and those that result in death [24]. A previous meta-analysis of four studies showed that elevated PCT was associated with a nearly five-fold increase in the risk of severe COVID-19(OR 4.76, 95% CI 2.74–8.29) [25]. Thus, it is of great significance to validate whether PCT is a reliable predictor of coinfections.

Our meta-analysis included five studies involving a total of 2775 patients, and the results showed that the ability of PCT to predict coinfections in patients with COVID-19 was limited (AUC = 0.72, SEN = 0.60, and SPE = 0.71). The results of three of the included studies suggested that PCT was a useful tool to rule out bacterial coinfections (its negative predictive value was over 93%) when its concentration was < 0.50 μg/L [13, 20, 21]. Notably, another study involving 2443 patients showed that PCT had a high negative predictive value of 95% [20] (Table 1).

This meta-analysis has several limitations. First, all included studies were retrospective except one, so the data were prone to confounding factors. Second, the included studies had a considerable level of heterogeneity. The number of included articles and the total number of patients were limited, so publication bias, subgroup, and sensitivity analyses could not be performed. More high-quality studies may be needed to elucidate the role of PCT in coinfections with COVID-19 and identify optimal cut-offs.

In summary, although PCT has a limited ability to diagnose coinfections in patients with COVID-19, low levels of PCT seem to be a good indicator for excluding coinfections. We remain skeptical about the ability of PCT to help clinicians detect coinfections early; more research is needed to validate the usefulness of PCT so that clinicians can initiate effective management quickly and reduce the overall mortality of COVID-19. Further research is needed to develop accurate predictive models and diagnostics for coinfections in patients with COVID-19.

## Supplementary Information

**Additional file 1**. Search strategy and results.

**Additional file 2**. Summary of the methodological quality of the studies according to the Quality Assessment of Diagnostic Accuracy Studies 2criteria.

## Data Availability

The raw data of this study are available from the corresponding author upon reasonable request.

## Abbreviations

PCT: Procalcitonin
COVID-19: Coronavirus disease 2019
CNKI: China National Knowledge Infrastructure
QUADAS-2: Quality Assessment of Diagnostic Accuracy Studies-2
SEN: Sensitivity
SPE: Specificity
AUC: Area under the curve
LRs: Likelihood ratios
DOR: Diagnostic odds ratio
SROC: Summary receiver operating characteristic
