# Supplementary material for: Predictive values of procalcitonin for coinfections in patients with COVID-19: a systematic review and meta-analysis
Source: Virol J. 2023 May 8;20:92. doi: 10.1186/s12985-023-02042-x (PMC10166029; doi:10.1186/s12985-023-02042-x)
Supplement: Supplementary file 1 — Additional file 1. Search strategy and results. [file 12985_2023_2042_MOESM1_ESM.docx]

**Search Strategy**

**Article title**: Predictive values of procalcitonin for coinfections in COVID-19 patients: a systematic review and meta-analysis

**Journal**: Virology Journal

**Authors**: Shanchen Wei^a^, Lina Wang^a^, Lianjun Lin^a^*, Xinmin Liu^a^*

^a^ Geriatric Department, Peking University First Hospital, Beijing 100034, China.

* Corresponding author at: Geriatric Department, Peking University First Hospital, Xishiku Avenue No 8, Xicheng District, Beijing100034, China.

Email addresses: 06474@pkufh.com (L.-J. Lin). lxm2128@163.com (X.-M. Liu).

ORCiD ID: 0000-0002-3264-5255 (X.-M. Liu).

**PubMed:**

| Search number | Query | Sort By | Filters | Results | Time |
| --- | --- | --- | --- | --- | --- |
| 4 | ((((((((((co-infection) OR (coinfection)) OR (super-infection)) OR (superinfection)) OR (secondary infection)) OR (bacterial infection)) OR (bacterial culture)) OR (other pathogens NOT SARS Cov-2)) OR (other organisms NOT SARS Cov-2)) AND ((procalcitonin) OR (PCT))) AND (((((Coronavirus disease 2019) OR (2019 Novel Coronavirus)) OR (SARS-CoV-2)) OR (2019-nCoV)) OR (COVID-19)) | | | 151 | 3:51:32 |
| 3 | ((((Coronavirus disease 2019) OR (2019 Novel Coronavirus)) OR (SARS-CoV-2)) OR (2019-nCoV)) OR (COVID-19) | | | 171,580 | 3:51:13 |
| 2 | (procalcitonin) OR (PCT) | | | 16,314 | 3:51:03 |
| 1 | ((((((((co-infection) OR (coinfection)) OR (super-infection)) OR (superinfection)) OR (secondary infection)) OR (bacterial infection)) OR (bacterial culture)) OR (other pathogens NOT SARS Cov-2)) OR (other organisms NOT SARS Cov-2) | | | 1,582,170 | 3:50:48 |

**Embase:**

| No. | Query | Results | Date |
| --- | --- | --- | --- |
| #11 | #1 AND #2 AND #10 | 285 | 2021/8/29 |
| #10 | #3 OR #4 OR #5 OR #6 OR #7 OR #8 OR #9 | 1185440 | 2021/8/29 |
| #9 | 'other organisms not sars cov-2' | 0 | 2021/8/29 |
| #8 | 'other pathogens not sars cov-2' | 0 | 2021/8/29 |
| #7 | 'secondary infection'/exp | 4595 | 2021/8/29 |
| #6 | 'bacterium culture'/exp | 108443 | 2021/8/29 |
| #5 | 'bacterial infection'/exp | 1071628 | 2021/8/29 |
| #4 | 'superinfection'/exp | 8844 | 2021/8/29 |
| #3 | 'coinfection'/exp | 51956 | 2021/8/29 |
| #2 | 'procalcitonin'/exp | 16504 | 2021/8/29 |
| #1 | 'coronavirus disease 2019'/exp | 142956 | 2021/8/29 |

**Web of Science:**

| No. | Query | Results | Date |
| --- | --- | --- | --- |
| #4 | ((#1) AND #2) AND #3 | 411 | 2021-8-29 4:44 PM |
| #3 | ((((((((TS=(co-infection)) OR TS=(coinfection)) OR TS=(super-infection)) OR TS=(superinfection)) OR TS=(secondary infection)) OR TS=(bacterial infection)) OR TS=(bacterial culture)) OR TS=(other pathogens NOT SARS Cov-2)) OR TS=(other organisms NOT SARS Cov-2) | 2875019 | 2021-8-29 4:44 PM |
| #2 | (TS=(procalcitonin)) OR TS=(PCT) | 51206 | 2021-8-29 4:41 PM |
| #1 | ((((TS=(Coronavirus disease 2019)) OR TS=(2019 Novel Coronavirus)) OR TS=(SARS-CoV-2)) OR TS=(2019-nCoV)) OR TS=(COVID-19) | 255206 | 2021-8-29 4:40 PM |

**Cochrane:**

| No. | Query | Results | Date |
| --- | --- | --- | --- |
| #1 | (Mesh descriptor: [COVID-19] explode all trees) OR (Mesh descriptor: [SARS Cov-2] explode all trees) | 527 | 2021-8-30 1:37 AM |
| #2 | (procalcitonin) OR (PCT) | 1842 | 2021-8-30 1:37 AM |
| #3 | (co-infection) OR (coinfection) OR (super-infection) OR (superinfection) OR (secondary infection) OR (bacterial infection) OR (bacterial culture) OR (“other pathogens NOT SARS Cov-2”) OR (“other organisms NOT SARS Cov-2”) | 38352 | 2021-8-30 1:40 AM |
| #4 | #1 AND #2 AND #3 | 6 | 2021-8-30 1:41 AM |

**CNKI:**

| No. | Query | Results | Date |
| --- | --- | --- | --- |
| #3 | (((主题：新型冠状病毒)OR(主题：新型冠状病毒肺炎))AND((全文：降钙素原)OR(全文：PCT)))AND((主题：双重感染)OR(主题：合并感染)OR(主题：细菌感染)OR(主题：细菌培养)OR(主题：继发感染)OR(主题：二重感染)OR(主题：重叠感染)） | 53 | 2021-8-30 2:22 AM |
| #2 | ((主题：新型冠状病毒)OR(主题：新型冠状病毒肺炎))AND((全文：降钙素原)OR(全文：PCT)) | 2621 | 2021-8-30 2:15 AM |
| #1 | (主题：新型冠状病毒)OR(主题：新型冠状病毒肺炎) | 342703 | 2021-8-30 2:14 AM |

**Wanfang:**

| No. | Query | Results | Date |
| --- | --- | --- | --- |
| #1 | 主题:(新型冠状病毒 or 新型冠状病毒肺炎) | 29704 | 2021-8-30 2:07 AM |
| #2 | 主题:(新型冠状病毒 or 新型冠状病毒肺炎) and 全部:(降钙素原 or PCT) | 292 | 2021-8-30 2:07 AM |
| #3 | 主题:(新型冠状病毒 or 新型冠状病毒肺炎) and 全部:(降钙素原 or PCT) and 主题:(细菌感染 or 细菌培养 or 合并感染 or 继发感染 or 双重感染 or 重叠感染 or 二重感染) | 44 | 2021-8-30 2:26 AM |
