# Supplementary figures and images for: Predictive values of procalcitonin for coinfections in patients with COVID-19: a systematic review and meta-analysis
Source: Virol J. 2023 May 8;20:92. doi: 10.1186/s12985-023-02042-x (PMC10166029; doi:10.1186/s12985-023-02042-x)

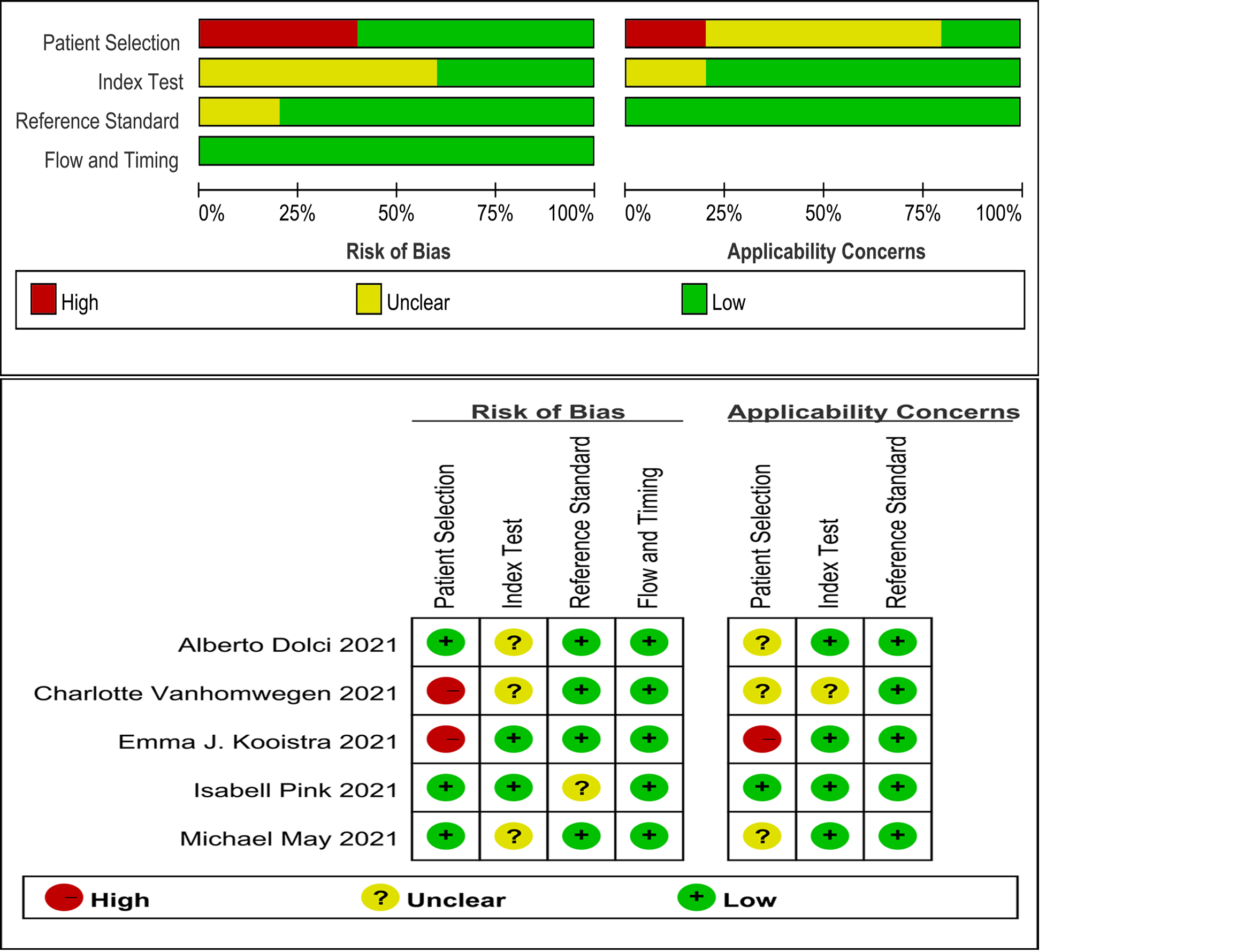

Supplement: Supplementary file 2 — Additional file 2. Summary of the methodological quality of the studies according to the Quality Assessment of Diagnostic Accuracy Studies 2criteria. [file 12985_2023_2042_MOESM2_ESM.tif]
